# Active link selection for efficient semi-supervised community detection

**DOI:** 10.1038/srep09039

**Published:** 2015-03-12

**Authors:** Liang Yang, Di Jin, Xiao Wang, Xiaochun Cao

**Affiliations:** 1 State Key Laboratory of Information Security, Institute of Information Engineering, Chinese Academy of Sciences, Beijing 100093, China; 2 School of Information Engineering, Tianjin University of Commerce, Tianjin 300134, China; 3 School of Computer Science and Technology, Tianjin University, Tianjin 300072, China

## Abstract

Several semi-supervised community detection algorithms have been proposed recently to improve the performance of traditional topology-based methods. However, most of them focus on how to integrate supervised information with topology information; few of them pay attention to which information is critical for performance improvement. This leads to large amounts of demand for supervised information, which is expensive or difficult to obtain in most fields. For this problem we propose an active link selection framework, that is we actively select the most uncertain and informative links for human labeling for the efficient utilization of the supervised information. We also disconnect the most likely inter-community edges to further improve the efficiency. Our main idea is that, by connecting uncertain nodes to their community hubs and disconnecting the inter-community edges, one can sharpen the block structure of adjacency matrix more efficiently than randomly labeling links as the existing methods did. Experiments on both synthetic and real networks demonstrate that our new approach significantly outperforms the existing methods in terms of the efficiency of using supervised information. It needs ~13% of the supervised information to achieve a performance similar to that of the original semi-supervised approaches.

In real life, the data of many complex systems are modeled as networks. Community structure, i.e., a group of densely connected subgraphs, has been shown as an important property of networks, although there is not a general and widely-accepted definition. Community detection becomes one of the most important tasks to explore and understand how the networks work. Since the emergence of this concept, a large number of community detection algorithms have been proposed^1^,^2^,^3^,^4^,^5^. Albeit some of them have achieved good performance, approaches based solely on network topology can not yield satisfactory results for the following two reasons. Firstly, the networks are often too complicated to be accurately detected. There usually exist overlapping communities or hierarchical structures in real-world networks. This makes it difficult to accurately determine community boundary and infer the number of communities. Secondly, the sparsity and noise of the topology also affect the precision of community detection^6^,^7^. Many nodes in the networks only have few noisy links, and hence it is unreliable to determine their affiliations based solely on topology.

To alleviate these aforementioned problems, some semi-supervised community detection algorithms have been proposed in the last few years^8^,^9^,^10^,^11^. By making use of the supervised information or background information, they significantly improved the performance of traditional topology-based methods. According to the types of the supervised information, semi-supervised community detection algorithms can be mainly divided into two categories. One group of them directly utilizes the node labels as the supervised information^9^,^10^. Although this is the most straightforward way to use supervised information, there exists a major drawback: community detection usually pays much attention to which nodes belong to the same group instead of the specific label of the community. Thus node label is not the most efficient supervised information in general. Furthermore, it is usually expensive to obtain the node labels in community detection. For example, in a social media networks, e.g., Facebook or Twitter, it is difficult to determine which community a person belongs to based on their profiles only. In contrast, it is much easier to determine whether two persons belong to the same community from their profiles. Thus, the other group of semi-supervised community detection algorithms utilizes the link information as the supervised information^8^,^11^,^22^. Using must-link and/or cannot-link information, the performance of community detection can be significantly improved, especially in networks with unclear structures. Link-based semi-supervised community detection methods usually focus on how to embed these supervised link information. They, however, often ignore the problem that which supervised link information is the most important and useful information for performance improvement, and they only add the randomly selected supervised link information. In fact, many nodes may have been correctly classified based on the topology, and it is wasteful and unhelpful to add the supervised link information relating to these nodes. Therefore, these current methods often need large amounts of supervised information to achieve the satisfactory performance.

In order to reduce the demand for the supervised information, we propose an active link selection framework which actively selects some links for human labelling. Motivated by active learning in machine learning which actively selects unlabelled data near the classification plane for human labelling, we find out that the nodes near the community boundaries are the most uncertain and informative ones and are critical for accurate community detection. The main idea is that, by connecting uncertain nodes to their community hubs and disconnecting the inter-community edges, one can sharpen the block structure of adjacency matrix more efficiently than randomly labeling links as the existing methods did. Specifically, this framework consists of three components. First, we conduct a nonnegative matrix factorization (NMF) process on the adjacency matrix of the original network. Second, we select some uncertain and informative links for human labeling based on the *Connection Strategy* (see Strategy 1). Third, since the added links indicate that their endpoints belong to the same community, they are more informative than the normal links in the original network. Therefore, to further utilize the added links, we perform the *Disconnection Strategy* (see Strategy 2). After these three steps, we obtain the new network topology, based on which we can conduct the next NMF process. This process is repeated until we obtain the satisfactory community results. The details of our two strategies are introduced as follows.

## Strategy 1 (Connection Strategy)

Based on the result of the current NMF, we select one hub (node with the smallest entropy) for each community; and select a set of inter-community links, whose endpoints both are on community boundaries (nodes with the largest entropy) and belong to different communities. Then, by human labelling if the two endpoints of a selected link actually belong to the same community, we will maintain this link; otherwise, we will disconnect this link. We also ask human to connect each endpoint to the hub of its own community.

Furthermore, we relax the Strategy 1 to ignore whether the endpoints of selected link belong to different communities. We name this relaxation of Strategy 1 as **Relaxed Strategy 1**.

## Strategy 2 (Disconnection Strategy)

Based on the result of the current NMF, we disconnect the links between the selected links' endpoints and the nodes which do not belong to the connected hubs' community. The intuitive idea is that, besides the hub, the endpoints of the selected link (to be labeled) may connect to other nodes which have different labels with the hub. Then, we have big reason to disconnect the links between the endpoints and these nodes.

Furthermore, the NMF algorithms are dependent on the initialization and need repeat on many different initializations, and iteratively conducting many different NMFs will be time-consuming. To solve this problem, we present a speedup scheme, i.e., taking the result of previous NMF as the initialization of next NMF process. The running time of the speedup scheme is shortened to one-twentieth of the original one. In addition, our framework is near linear with the size of the network, and can easily be parallelized and distributed (see the “Complexity analysis and scalability” section). Thus, it has the potential to be applied to real large-scale problems.

To our knowledge, we are the first to design active link selection strategy for community detection. Although there have been some previous works on using active learning in community detection, they all focus on active node selection rather than link selection^12^,^13^. We summarize the main contribution as designing an active link selection framework as well as its speedup scheme for effective and efficient semi-supervised community detection.

## Results

In this section, we demonstrate the effectiveness and efficiency of our proposed active link selection framework for semi-supervised community detection. To this end, we apply it on two types of synthetic datasets and six widely-used real networks. To verify the efficiency of the proposed framework, we take a recently proposed semi-supervised community detection method^22^ as the baseline. This method randomly selects two nodes in the network, and asks a human if these two nodes belong to the same community. If the answer is “yes”, this method adds an edge between them or increases the edge weight. If the answer is “no”, it removes the edge between these two nodes or decreases the edge weight. Thus, this method encodes the labels of the links which are randomly selected without taking into account the performance impact of the selected links. Since there are three different strategies in our framework, to illustrate the details of each strategy on performance improvement, we test on four strategy combinations: only Connection Strategy (S1), only relaxed Connection Strategy (rS1), Disconnection Strategy on top of Connection Strategy (S12) and Disconnection Strategy on top of relaxed Connection Strategy (rS12). To quantitate the performance, we make use of normalized mutual information (NMI)^14^ to evaluate the performance of community detection. Compared to the ground-truth community label *C_g_*, the NMI of the resulted community label *C_r_* from algorithm is:
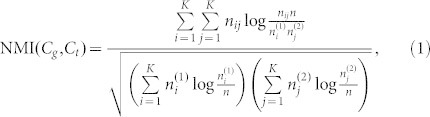
where *K* is the number of communities, *n* the number of nodes, *n_ij_* the number of nodes in ground-truth community *i* that are arranged to the resulted community *j*, *n_i_*^(1)^ the number of nodes in ground-truth community *i* and *n_j_*^(2)^ the number of nodes in resulted community *j*. Besides, we can use the growth rate of NMI as a function of the percentage of the added and removed links to measure the efficiency of different strategy combinations.

### Synthetic networks

**Figure 1:**
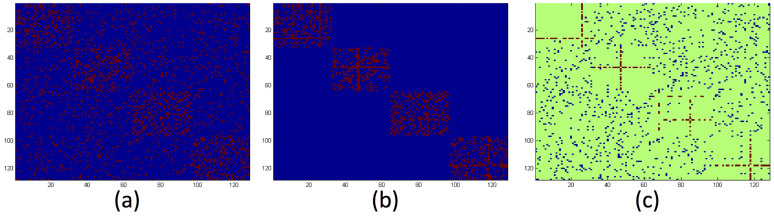
Adjacency matrix changes. (a) the original adjacency matrix of the GN network with *Z_out_* = 8, (b) the final adjacency matrix after 50 iterations, and (c) the differences between the final and original adjacency matrices in which the red and blue elements denote the added and removed edges, respectively.

Firstly, we apply our approach to two types of synthetic network datasets, i.e., Girvan-Newman (GN) benchmark networks^4^ and Lancichinetti-Fortunato-Radicchi (LFR) benchmark networks^15^. Each GN network is divided into four non-overlapping communities each of which has 32 nodes. Each node has 16 edges on average and connects to *Z_in_* nodes in its own community and *Z_out_* nodes in other communities. The community structure is clear when *Z_out_* is small (*Z_out_* < 5). As *Z_out_* increases, however, the community structure becomes vague and the community detection becomes challenging. In Figure 1, we give a demonstration to show how our framework sharpens the block structure of the network adjacency matrix. As shown in Figure 1(a), the block structure of the community in GN network with *Z_out_* = 8 is not clear since there are too many non-diagonal elements, which means there exist many inter-community connections. As shown in Figure 1(b) and (c), by connecting some uncertain nodes with the hubs in their own communities (Connection Strategy) and disconnecting them with the nodes, which do not belong to the communities of the connected hubs (Disconnection Strategy), the block structure becomes very clear and the community detection task becomes much easier.

**Figure 2:**
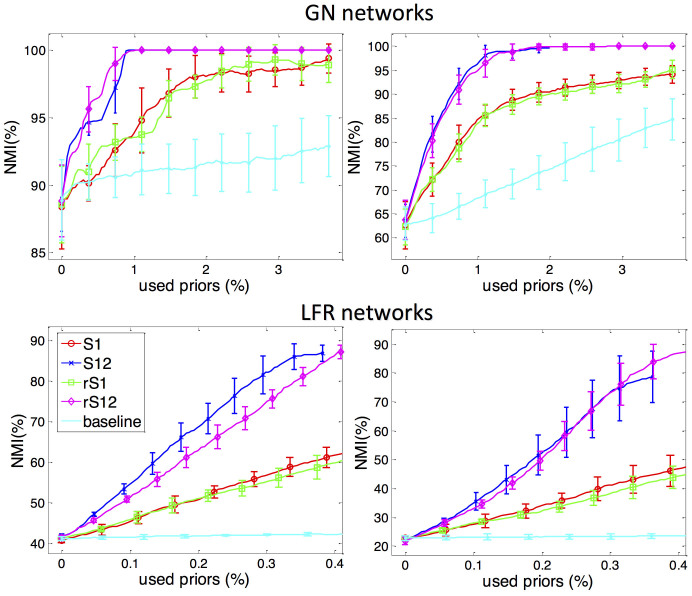
The performance of active link selection framework on synthetic networks. Each curve and error bar represent the average and standard deviation of ten trials, respectively. The figure (a) and (b) are the results from GN networks with mixing parameter Z_out_ = 7 and 8, respectively, while the figure (c) and (d) are the results from LFR networks with small community size (10–20) and large community size (20–100), respectively.

As shown in the first row of Figure 2, compared with the baseline, all the four strategy combinations can significantly improve the community detection performance with the same amount of supervised information. For example, in GN networks with *Z_out_* = 8, the performance can be improved to over 85% and 95% from 62% by adding 1% labelled links using only Connection Strategy and both Connection Strategy and Disconnection Strategy, respectively. In contrast, the performance of baseline method only achieves 68% by adding 1% randomly selected labelled links. The performance improvement using both the two strategies is ~5 times than that using the baseline method.

To further demonstrate the efficiency, we apply our approach to LFR benchmark networks^15^. Comparing with GN networks, LFR networks are more complex and challenging. In LFR networks, the distributions of node degree and community size obey power laws with parameters γ and β. At the same time, we can specify the number of nodes, the fraction of inter-community edges (mixing parameter μ), minimum and maximum community size. In this paper, we follow experiment setting design by Lancichinetti: set the number of nodes to 1000, the minimum community size to 10 or 20, the maximum community size to 5 times the minimum community size, the exponent of node degree distribution and community size distribution to 2 and 1, respectively. We vary the mixing parameter from 0.65 to 0.8, since it plays a major role in determining the clarity of community structure as *Z_out_* in the GN networks. In the second row of Figure 2, we plot the performance improvement using different link selection strategies on LFR networks with different community sizes and mixing parameters. We find out that our two proposed strategies significantly and efficiently improve the community detection performance, especially on networks with large mixing parameter and/or large community size. Taking networks with mixing parameter μ = 0.75, minimum and maximum community size *c*_min_ = 20, *c*_max_ = 100 as an example, the performance can be improved to 60% and 86% from 27% by adding 0.4% labelled links using only Connection Strategy and both Connection Strategy and Disconnection Strategy, respectively; while the performance reaches only 29% when using the baseline method. These experiments further illustrate the effectiveness and efficiency of our proposed active link selection framework on synthetic networks.

### Quantitative comparisons on real-world networks

In this section, for quantitative comparison, we apply our proposed active link selection on 18 widely-used real world networks, as shown in Table 1. Herein Friendship6 and Friendship7 are the same network with different ground-truth communities. The Friendship6 classifies students into different communities based on their grades, while the Friendship7 divides the grade 9 into two communities, i.e., the white students and the black students.

**Figure 3:**
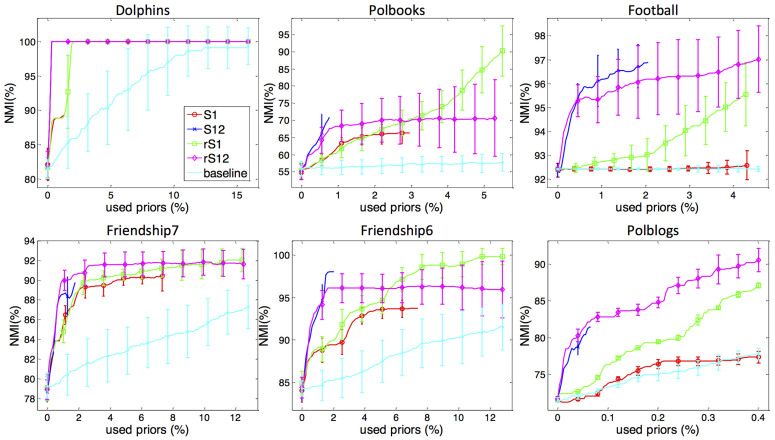
The performance of active link selection framework on 6 real networks. Each curve and error bar represent the average and standard deviation of ten trials, respectively. The cyan line is the result of baseline method, which adds random selected links. The red and blue lines are the results of our framework using only Connection Strategy and relaxed Connection Strategy, respectively. The green and magenta lines are the results of our framework using Disconnection Strategy on top of the Connection Strategy and relaxed Connection Strategy, respectively.

First of all, we choose 6 classic networks, i.e. Dolphins, Polbooks, Football, Friendship6, Friendship7 and Polblogs, for detailed comparison. The size of these networks is less than 1500, and we repeat the experiments 10 times with different initializations. In Figure 3, we provide the performance of above 4 strategy combinations and the baseline method on six real-world networks. Each curve is the performance obtained when different percentages of labelled links are added. It is easy to find out that all the 4 strategy combinations grow much faster than the baseline method on each of the networks. Furthermore, S12 and rS12, which are the combination of connection and disconnection strategy, grow faster than the S1 (and rS1) which only make use of the Connection Strategy (and its relaxed version). Taking Dolphins Social Network as an example, the NMI index of NMF based on the original topology only achieves ~82%. To achieve 100%, the rS12 and rS1 need 0.5% and 2.5% labelled links, respectively, while the random semi-supervised method needs more than 15% labelled links. Taking the School Friendship Network as another example, if the number of communities is 6, by adding 5% labelled links, S1 and S12 achieve 86% and 90%, respectively; while the baseline method only achieve less than 80%. If we take 7 as the ground-truth number of communities, in order to achieve 92%, rS1 and rS12 need less than 3% and 1% labelled links, while the baseline method need much more than 13% labelled links.

Although the combinational strategies have the fastest growth rate at the beginning period of adding labelled links, they keep a stable status when the number of added links achieves a certain threshold on Small and medium scale networks. The reason may be that, although Disconnection Strategy disconnects many inter-community links, since we use the results from the previous NMF, it may also disconnect some intra-community links. Although we can solve this problem by using the ground-truth labelled links instead of the results from last NMF, this solution dramatically increases the demand for supervised information, which is contrary to our original intention. In addition, although the standard Connection Strategy is more intuitive, the relaxed Connection Strategy achieves the similar or even better performance than the standard one, since the answer to whether two nodes belong to different communities is also from the result of the previous NMF instead of the ground-truth labels.

**Figure 4:**
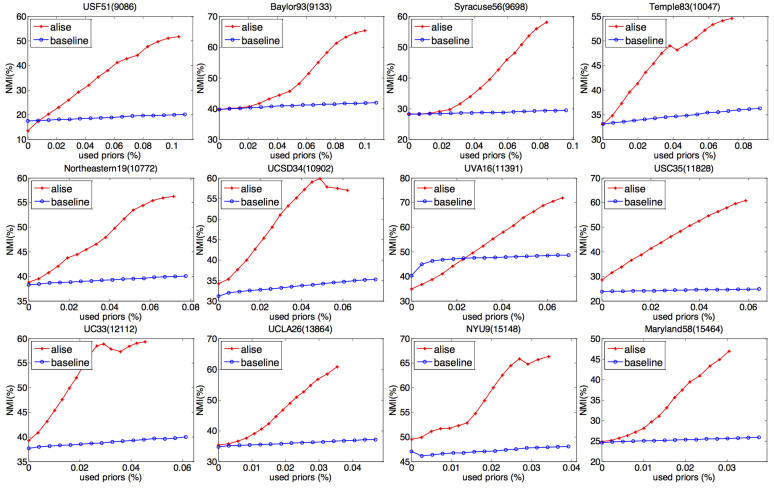
The performance of active link selection framework on 12 Facebook networks at United States universities. In each figure, the blue line is the result of baseline method, while the red line is the result of our framework using Disconnection Strategy on top of the relaxed Connection Strategy.

To further validate the framework, we compare the performance of our active link selection framework and the baseline method on 12 large-scale social networks, the sizes of which are in the range of 12,000 to 21,000 (see Table 1). These networks are Facebook social networks at different universities in U.S. The friendships are undirected, and there are six pieces of person's metadata which are *residence hall*, *major*, *second major*, *class year*, *former high school* and *gender*, respectively. As suggested by Traud *et al*.^24^, we use the class year as the ground truth community. For simplicity, we only compare the performance of the rS12 (i.e., Disconnection Strategy on top of the relaxed Connection Strategy) with that of the baseline method, as shown in Figure 4. It is easy to find out that, the performance of the original NMF-based community detection method only achieves less than 40% on most of the networks (except for *NYU* network), and the performance improvement is very small by adding the randomly selected supervised link information as the baseline method did. On the other hand, by adopting our proposed framework this performance can be significantly improved. Taking *Northeastern* network as an example, the performance of the NMF-based method achieves 38.2%. By adding 0.03%, 0.04%, 0.05% 0.06% and 0.07% supervised link information respectively, the baseline method which randomly selects the supervised link information only achieves 39.00%, 39.27%, 39.48%, 39.75% and 39.99%; while our active link selection framework achieves 45.77% 48.70%, 52.82%, 55.09% and 56.19%, respectively. The performance improvement of our framework is ~9 times that of the baseline method.

In summary, from quantitative comparison, we find out that our proposed active link selection framework needs much fewer supervised information than the state-of-the-art random semi-supervised community detection methods to achieve the same performance. In other words, the proposed active link selection framework is more efficient in the use of supervised link information.

### Case studies on real-world networks

In this section, we choose 3 networks for case studies and one of them, i.e., the Dolphins Social Network, for detailed illustration.

**Figure 5:**
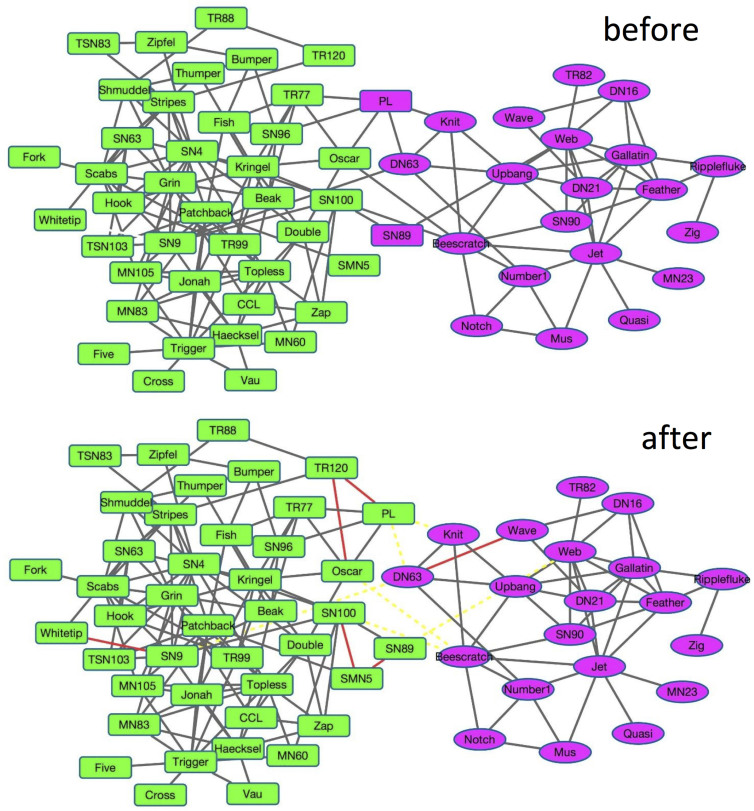
Topologies and detected communities of Dolphin network before and after using the active link selection. The shape *square* and *circle* represent the ground-truth communities are *female* and *male* dolphins, respectively. And the color *green* and *purple* represent the estimated communities are *female* and *male* dolphins, respectively. In the figure, the red lines denote added must-link and the yellow dashed lines denote the removed links.

As shown in Figure 5(a), the Dolphins Social Network^16^, which was reported by Lusseau, is an undirected social network between 62 dolphins. If two dolphins are together more often than expected by chance, there exists a link between them. These dolphins are divided into two communities based on their genders, i.e., male dolphins (cycle nodes) and female dolphins (rectangle nodes).

As shown in Figure 5, before using our proposed active link selection framework, nonnegative matrix factorization (NMF) method based on original network topology cannot correctly classify dolphins *FL* and *SN89* as male dolphins, and the NMI of it only achieves ~81%.

Under our framework, we firstly select one hub for each community based on the entropy of nodes. *TR120* is selected as the hub of female dolphin community, while *Wave* is selected as the hub of male one. We conduct the first round active link selection based on the result of NMF. According to Strategy 1, we select the link between dolphins *PL* and *Oscar*, which is the link with the largest entropy, for human labelling. Since these two nodes belong to the same community, we keep this link, and connect them to hub of female community, i.e., *TR120*, according to human labels. According to Strategy 2, we remove the links <*PL*, *Knit*>, <*PL*, *DN63*> and <*Oscar*, *Beescratch*>, since *Knit*, *DN63* and *Beescratch* does not belong to the community of *TR120* based on the result of NMF. Having obtained the new network topology, we run the second NMF based on the new topology. From the result we find out that dolphin *PL* can be correctly classified and the NMI achieves 89%. Thereafter, we conduct the second round active link selection on the result of the second NMF. As before, we select link between dolphins *SN100* and *SN89* for human labelling. According to the labelling results, we keep this link, connect them to new hub *SMN5* and disconnect link <*SN100*, *Beescratch*> and <*SN89*, *Web*>. Similarly, based on the new network topology, we run the third NMF. It correctly classifies all the dolphins, and the NMI achieves 100% now. If we repeat this process, link <*SN9*, *DN63*> will be selected, and corresponding links will be added and removed. However, it will not affect the final community detection result any more. The final network topology and community detection result after using our framework are shown in Figure 5. In the figure, the red lines denote added must-link and the yellow dashed lines denote the removed links. From the final network topology, we find out that active link selection framework separates dolphin nodes into two disconnected subgraphs, which makes community detection much easier. From this visualization, we find out that the proposed strategies can not only select the most uncertain nodes which significantly affect the detection performance, but also correctly classify them by using only a few human labelled links.

The American College football network^4^, which was compiled by Girvan & Newman, is the network of American football games between Division IA colleges during regular season Fall 2000. There are 115 terms represented as nodes, which belong to 12 different conferences. Terms have a link with each other if they played against in that season. Most terms frequently played against other terms in the same conference.

**Figure 6:**
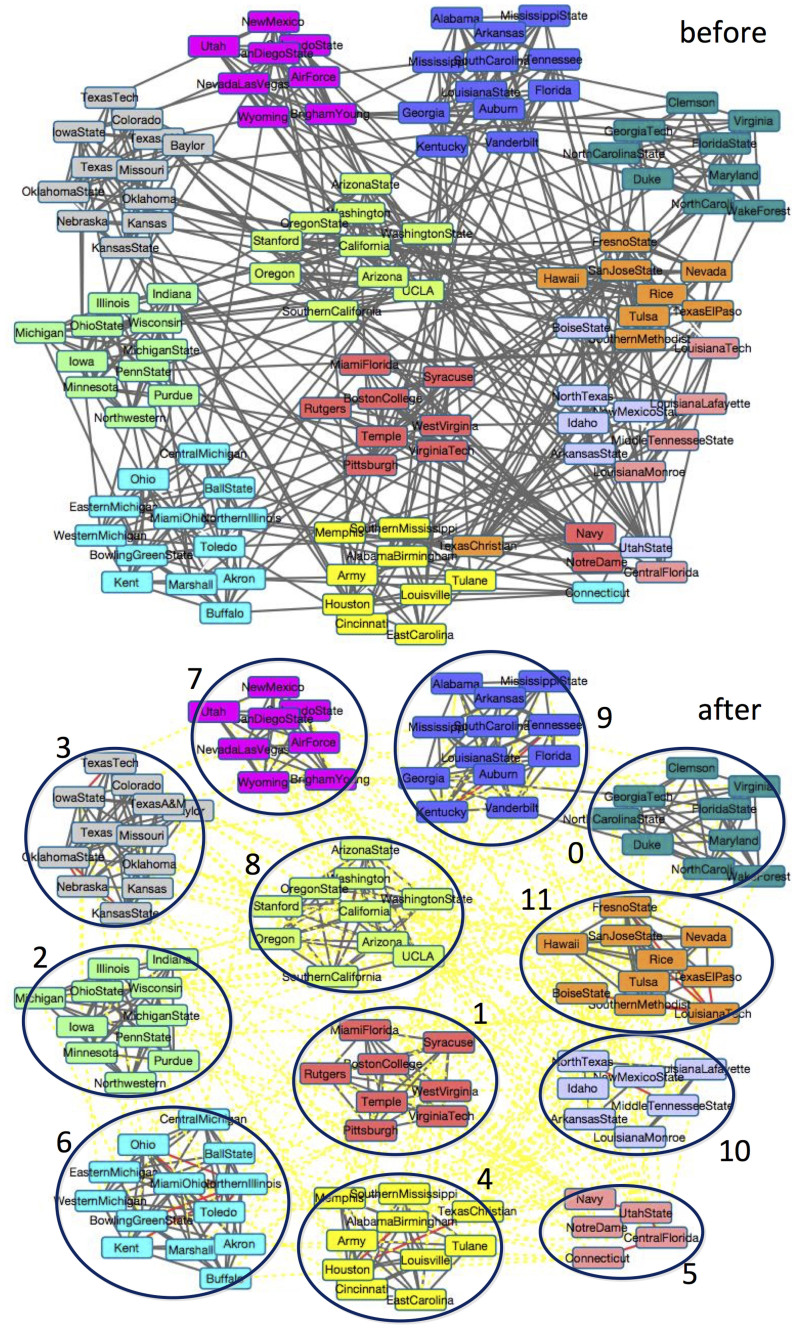
Topologies and detected communities on network Football before and after using the active link selection. The color represents the estimated communities. In the figure, the red lines denote added must-link and the yellow dashed lines denote the removed links.

As shown in Figure 6, by setting the community number *K* = 12, NMF method can correctly classify 8 communities based on original network topology. In conference 4 (*Conference USA*), there is only one node (*TexasChristian*), which is not correctly classified, since it played against more teams in conference 11 (*Western Athletic*). There are 8 teams from conference 11 in all the 11 teams it has played against. However, most of the nodes in conferences 5 (*Independents*) and 10 (*Sun Belt*) are misclassified for the following two reasons. Firstly, some nodes play against teams in other conference more frequently than those in their own, such as teams *Navy* and *NotreDame* in conference 5. From the viewpoint of community detection, this means there are too many inter-community edges. Secondly, there are some subgroups in a conference, and teams only play against others in their own subgroup. This makes one community be divided into some small communities. For example, conference 10 is divided into two small conferences. From the perspective of community detection, this community is lack of sufficient intra-community edges.

As shown in Figure 6(b), by iteratively processing active link selection 50 times, i.e., adding or removing 150 links, most of the inter-community links are removed and some important intra-community links are added. Taking term *BoiseState* as an example, since it only played against 9 terms in other conferences, it is very difficult to correctly classify it. By connecting it with term *LouisianaTech* which is the hub of conference 11 and disconnecting links with the terms from other conferences, term *BoiseState* can be correctly classified into conference 11. Compared with the random semi-supervised method proposed by Zhang^22^, which needs adding 1199 labelled links to correctly classify all the nodes, our proposed active link selection framework only needs less than 150 labelled links to achieve the same performance. This demonstrates that our actively selected links are more critical than randomly selected links and the use of the labelled links is more efficient.

The School Friendship Network^17^, which was compiled from the National Longitudinal Study of Adolescent Health, is based on self-reporting from students whose grades range from 7 to 12. According to the different grades, the number of communities is 6. However, there are two factors, which make the accurate detection difficult. Firstly, there are only 4 students (1, 2, 68 and 69) in grade 12, and they do not densely connect with each other. Secondly, in grade 9, there are two subgroups corresponding to white and black students.

**Figure 7:**
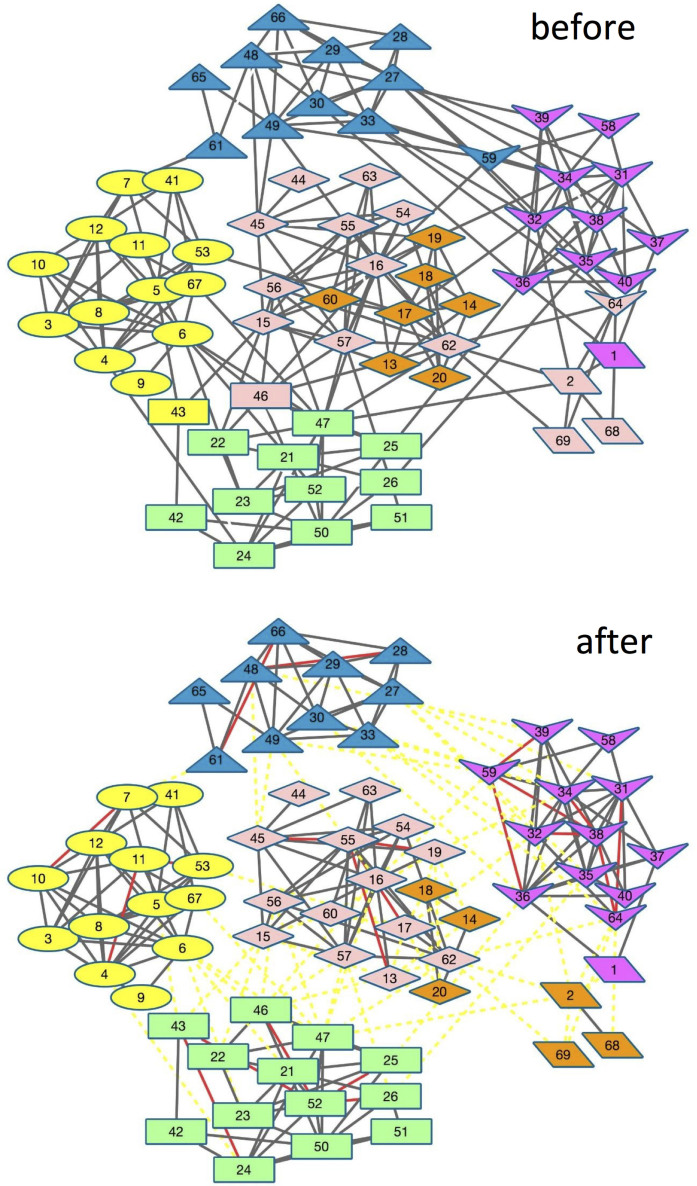
Topologies and detected communities on Friendship 6 network before and after using the active link selection. The shape represent the ground-truth communities, while the color represents the estimated communities. In the figure, the red lines denote added must-link and the yellow dashed lines denote the removed links.

As shown in Figure 7(a), by setting community number *K* = 6, NMF method can neither correctly classify students in grade 9 and 12 nor correctly classify students near the community boundaries. On one hand, it misclassifies students near the community boundary, i.e., 43, 46, 59 and 64. On the other hand, it divides the grade 9 into two groups, and one of them is merged with the grade 12. As shown in Figure 7(b), by using our proposed active link selection strategy and labelling only 42 links, the above-mentioned two problems are significantly alleviated. Firstly, all the students near the community boundary are correctly classified. For example, since students 43 and 46, which belong to grade 8, connect with much more students in other grades, they are misclassified using original topology-based method. In contract, by connecting them with the hub of grade 8 (student 52), they are correctly classified based on the modified topology. Besides, by disconnecting some links between grade 9 and grade 12, we can correctly distinguish these two grades, although there remain some nodes in grade 9 are classify into grade 12, i.e., students 14, 18 and 20. The cause of this phenomenon is that there does not exit any hub in grade 12, which makes us cannot add some must-links between hub and the nodes in grade 12. As shown in next section, we will find out that our proposed framework can correctly classify all the students by setting community number *K* = 7 when using only Strategy 1.

To sum up, these above 3 examples all demonstrate the high efficiency of our proposed active link selection framework. This mainly stems from both the high efficient links selection strategy and high efficient use of labelled information. First, Strategy 1 can select the most critical links for human labelling. Most of these selected links are near the community's boundaries. Second, Strategy 2 can further make full use of the added links to disconnect the inter-community edges. Although there are few wrong disconnections, the majority of the disconnected links are important for enhancing the performance. As shown in above four figures, most of the removed links, which are represented as yellow dashed lines, play an adverse role in accurate community detection.

### Parameters setting

**Figure 8:**
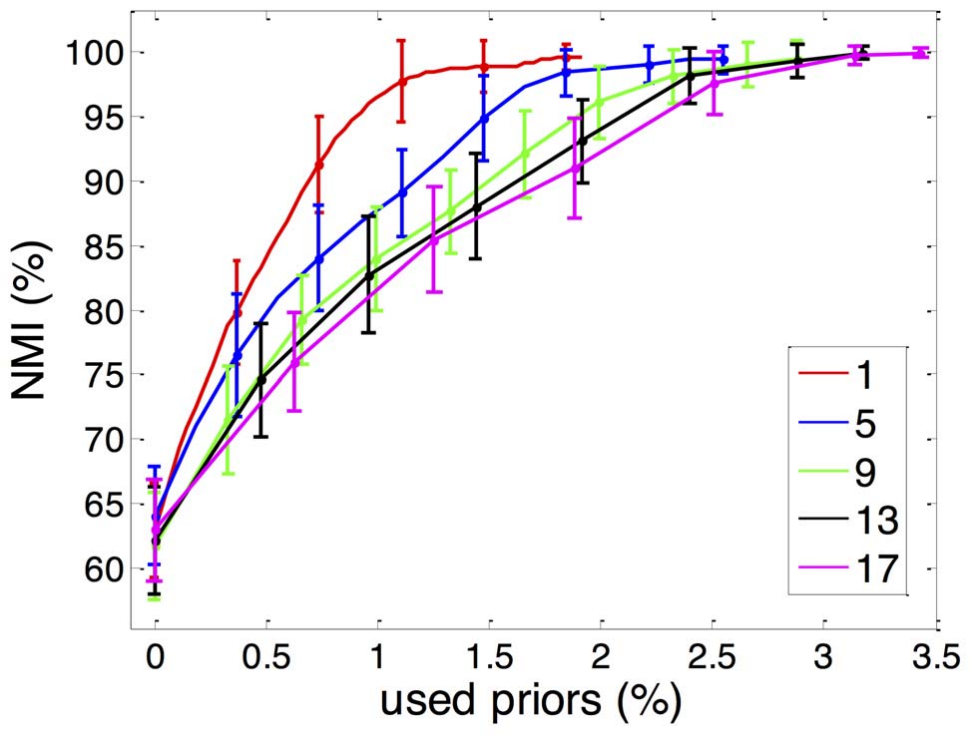
The impact of parameter *n_select_* on the performance improvement.

In our proposed active link selection framework, there are two parameters to be determined. The first is the number of selected links in Connection Strategy *n_select_*, and the second is the number of iterations *n_iter_*. To investigate the impact of *n_select_* on performance improvement, we conduct parameter tuning on GN (Girven-Newman) networks, and vary *n_select_* from 1 to 20. As shown in Figure 8, as the *n_select_* increases, the growth rate slightly decreases. Thus, we should give priority to small *n_select_*. Besides, we need to perform an accelerated NMF in each iteration, which will take much more time on large networks. Balancing the growth rate and running time, we set the *n_select_* as the one tenth of the number of nodes, i.e., *n_select_* = 0.1*n*. For the number of iterations *n_iter_*, we determine it by stopping the iteration when there are 5 iterations without membership changes.

## Discussion

In this paper, we propose an active link selection framework for efficient semi-supervised community detection. This framework consists of two components: Connection Strategy and Disconnection Strategy. By using the Connection Strategy, we interactively select uncertain links for human labeling based on the result of nonnegative matrix factorization. To further exploit the added links, we design the Disconnection Strategy to disconnect the inter-community links. These two strategies together can efficiently sharpen the block structure of the network's adjacency matrix. Then based on the labelled links, we modify the network topology and perform the next nonnegative matrix factorization. Compared with the traditional semi-supervised methods which add the randomly selected supervised information, our framework actively selects uncertain links for labelling, which significantly reduces the demand for supervised information and obviously improves the efficiency of the semi-supervised community detection. Besides, we proposed a speedup scheme to accelerate the NMF over the iterations tenfold. The experiments on two types of synthetic benchmarks and several widely-used real networks demonstrate the effectiveness and efficiency of our approach.

There remain some interesting problems related to our work. In the current work, we should predefine the number of communities as a prior. However, as we added labelled links, our framework sharpens the block structure of the networks, which indicates the number of communities. In future work, we want to further investigate how to determine the number of community and improve the detection performance simultaneously. Besides, whether combining the active link selection and the active node selection can further improve the efficiency of the semi-supervised community detection is also what we are interested in in the future.

## Methods

In this section, we first give an overview of our proposed active link selection framework. Then, we describe the generative model of community detection, which can be formulated as a nonnegative matrix factorization (NMF) problem, and present the details of our active link selection strategies based on the result of the NMF formulation. Finally, we provide a speedup scheme for NMF in our framework.

### Overview

**Figure 9:**
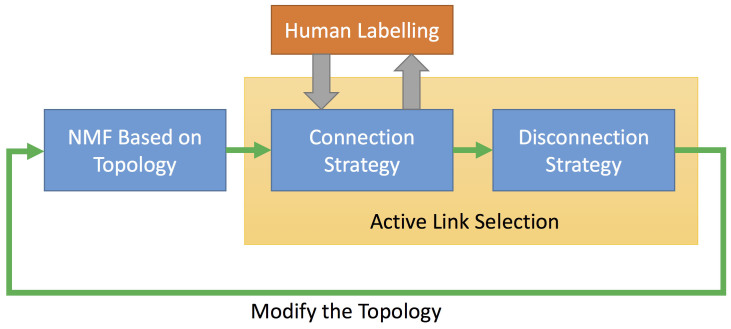
The workflow of the active link selection framework.

In Figure 9, we show the flow chart of our active link selection framework. In each iteration, we first conduct a NMF according to network topology. Based on the result of the current NMF, we then apply Strategy 1 (Connection Strategy) to actively select the most uncertain links for human labelling. Notice that this is an interactive process instead of just the passive acceptance of labelled links. To further exploit the obtained label information, we apply Strategy 2 (Disconnection Strategy) to disconnect inter-community links that may affect the performance. Notice that Strategy 2 does not require human involvement any more. According to the results from above two strategies, we modify the network topology, which is the input of the NMF in next iteration. This iteration is stopped when there are 5 iteration steps without membership changes.

### Generative model

A network can be modelled as a graph *G* = (*V, E*), in which *V* is a set of *n* nodes and *E* a set of *m* edges each of which connects two nodes in *V*. For simplicity, we assume *G* is an undirected and unweighted graph whose adjacency matrix can be represented as a nonnegative symmetric binary matrix **A**. The element *a_ij_* = 1 if and only if there exists an edge between nodes *i* and *j*, and *a_ii_* = 0 for any 1 ≤ *i* ≤ *n*. Besides, we assume there are *K* communities in the network, which is known as prior information.

In the generative process of the network^18^, *a_ij_* is an observed variable which denotes the probability of existing a connection between nodes *i* and *j*, and it is determined by the probability that they belong to the same community. We define the soft membership indicator variable *x_ik_* as the probability that node *i* belongs to community *k*. Under this model, an expected edge *<i,j>* can be generated in the following steps. First we uniformly select a community *k*. And then community *k* selects a pair of nodes, i.e., nodes *i* and *j*, with the probability *x_ik_* and *x_jk_*, respectively. Finally these two nodes form an edge in community *k*. Summing over all the communities *k*, the expected number of edge between nodes *i* and *j* can be formulated as
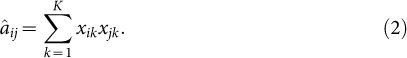


Using the matrix form, the above formula can be rewritten as
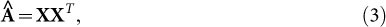
in which 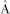 denotes the expected adjacency matrix, and **X** is the soft membership indicator matrix. As a result, we transform the community detection problem into a nonnegative matrix factorization (NMF) problem. We make use of square loss function to measure the difference between the observed edge and the expected number of edge, and formulate the community detection problem as the following optimization problem
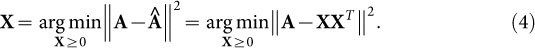


By using gradient descent method introduced by Wang *et al.*^19^, we can obtain the multiplicative update rule for element *x_ik_*as
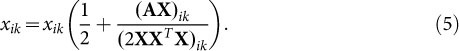


By normalizing, the *i^th^* row of **X**, i.e., ***x****_i_*_,_ can be seen as the membership probability distribution of node *i*. One can classify node *i* to community *k* if *x_ik_* is the largest element in vector ***x****_i_*.

### Active link selection strategies

Since each row of the community membership **X** indicates the membership probability distribution of the corresponding node, we use it as the evidence to find the hubs and the most uncertain nodes in each community. To design a quantitative measure to select the community hubs and the most uncertain nodes, we make use of entropy (information entropy) of the membership probability distribution:
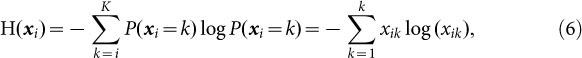
as the criterion. Entropy is a measure to characterize the uncertainty of an event, being larger for more random event. For example, if the probabilities that one node belongs to *k* different communities are equal, i.e., 1/*k*, the entropy of this node is the largest. In contrast, if the probability that one node belongs to a specific community is 1, and the probability that it belongs to other communities is 0, the entropy of that node is the smallest. Having defined the entropy of a node, the entropy of a link can be defined as the average of the entropies of its two endpoints. To facilitate description, we provide the following two definitions.

**Definition 1:** The hubs of a community are defined as the set of nodes belonging to this community and having the smallest entropy.

**Definition 2:** The boundary nodes of a community are defined as the set of nodes belonging to this community and having the largest entropy.

As shown in the “Results” section, there are two main reasons why many topology-based community detection methods degrade or fail. The first one is that there exist many inter-community edges, which seriously break the block structure of the network's adjacency matrix. Most endpoints of these edges are on the boundaries of communities. They are the most uncertain nodes, and are likely to be misclassified. Thus, what we should firstly verify using human labelling is the inter-community edges, whose endpoints are both on the community boundaries. In order to efficiently improve the performance of community detection, human only needs to label the links by deciding whether these two nodes belong to the same community rather than the specific community of each node.

**Figure 10:**
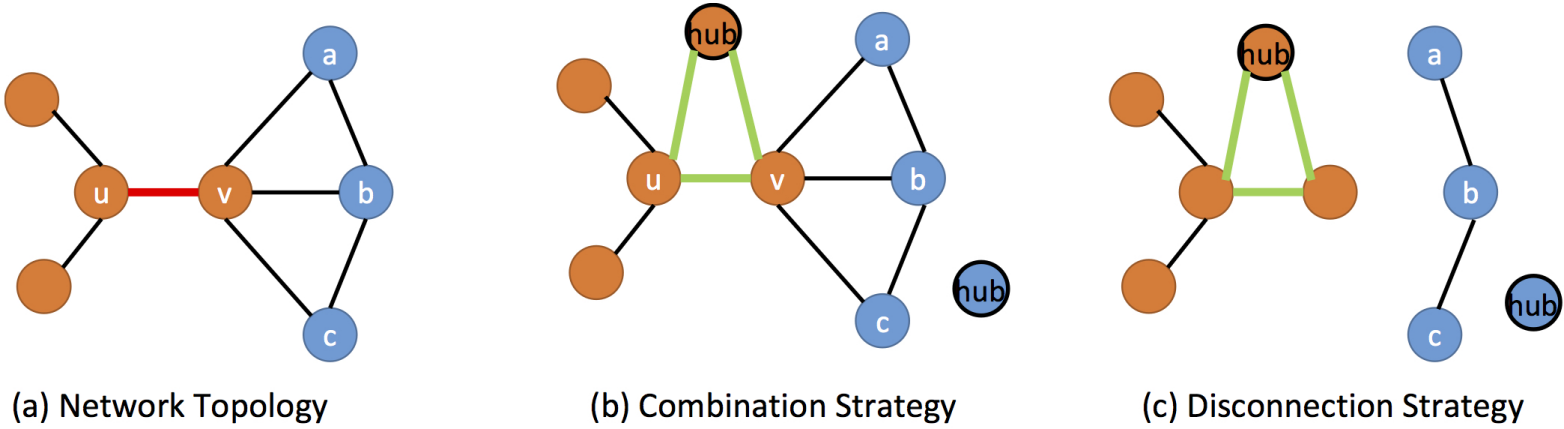
The visual explanation of the two active selection strategies. (a) Network topology and selected link. (b) Connection Strategy. (c) Disconnection Strategy.

In addition, another reason why nodes are misclassified is that nodes may connect with more nodes in other communities than in its own. Taking the network in Figure 10(a) as an example, nodes *u* and *v* belong to the community marked as “orange”, but node *v* connects with much more nodes in the community marked as “blue”. Topology-based methods usually classify nodes *u* and *v* into the “orange” and “blue” community, respectively. Even though the link <*u*,*v*> is selected as the inter-community edge for human labelling, we also cannot correctly classify node *v* into the “orange” community. To solve this thorny problem, after each NMF process, we select a hub for each community, and ask human to connect each endpoint to the hub of its own community. As before, human only needs to decide whether two nodes belong to the same community. As shown in Figure 10(b), by connecting nodes to their corresponding hubs, node *v* will be drawn toward the “orange” community. Although we can enhance the impact of added links by imposing large weights on them, we set the weights of all the links to 1 for simplicity. We summarize this connection strategy as follows:

### Strategy 1 (Connection Strategy)

Based on the result of the current NMF, we select one hub (node with the smallest entropy) for each community; and select a set of inter-community links, whose endpoints both are on community boundaries (nodes with the largest entropy) and belong to different communities. Then, by human labelling if the two endpoints of a selected link actually belong to the same community, we will maintain this link; otherwise we will disconnect this link. We also ask human to connect each endpoint to the hub of its own community.

Furthermore, since we determine whether the endpoints of selected link belong to same community only based on the result of NMF instead of the ground-truth. This decision may be wrong. Thus, we relax the Strategy 1 to ignore whether endpoints of selected link belong to different communities. We name this relaxation of Strategy 1 as **Relaxed Strategy 1**.

To further exploit the added links between endpoints and hubs, we also disconnect the links between the endpoints and other nodes, which do not belong to connected hubs' community. The intuitive idea is that, besides the hub, the endpoints of the selected link (to be labeled) may connect to other nodes which have different labels with the hub. Then, we have big reason to disconnect the links between the endpoints and these nodes. As shown in Figure 10(c), since nodes *a*, *b* and *c* do not belong to the community of *v*'s connected hub, the links between *v* and nodes *a*, *b*, *c* are disconnected. We should notice that, although by asking human to label these links we can obtain completely accurate results, it may need large amounts of human involvement. Since the aim of our proposed framework is to reduce the demand for supervised information, here we only use the result of last NMF as the evidence to disconnect links. Though it may introduce some wrong disconnections, the experimental results show that this scheme can significantly improve the performance of community detection. We summarize this disconnection strategy as follows:

### Strategy 2 (Disconnection Strategy)

Based on the result of the current NMF, we disconnect the links between the selected links' endpoints and the nodes which do not belong to the connected hubs' community.

By using connection strategy, we can interactively select the most uncertain links for human labelling based on the result of the NMF. In addition, by adopting disconnection strategy, we can make full use of the information inherent in the added links. These two strategies both significantly and efficiently improve the performance by sharpening the block structure of the network's adjacency matrix, i.e., disconnecting the inter-community links and connecting uncertain nodes with the hub of its own community.

### Speedup Strategy

As mentioned by Wang *et al.*^19^, since the NMF problem in equation (4) is not convex, the result of gradient descent is dependent on the initialization. Thus, to obtain the satisfactory result, we usually repeat the iterative updating rule in (5) using many different initializations (20 times in general) and choose the result with the smallest error. This will reduces efficiency of the nonnegative matrix factorization algorithm. Besides, since we need iteratively solve many different NMFs, this problem becomes much more serious in our framework. From experiments, we find out that the two consecutive NMFs in our framework often take the similar network topologies as input, since we only modify the edges related to 2*n_select_* nodes. Thus their results should be similar. So we can use the result of the previous NMF as the initialization of the current one. This makes our algorithm more efficient for following two reasons. On one hand, the initialization of NMF is near the final result, so the updating rule only needs few steps to converge to minima. On the other hand, we do not need repeat it using different initializations. As a result, our accelerated algorithm only spends less than one twentieth times than before, and it is also very important on real large networks.

### Complexity analysis and scalability

By taking into account the sparsity of the adjacency matrix **A**, the complexity of each non-negative matrix factorization iteration is O((*nK + m*)*K*). Connection Strategy needs O(*n + m*) floating-point operations to compute the entropy of nodes and edges, O(*pm*) floating-point operations to select *p* most uncertain links, and O(*n*) floating-point operations to select one hub for each community. Thus, the complexity of the Connection Strategy is O(*n + pm*). The Disconnection Strategy needs O(*pd*) floating-point operations to disconnect the most likely inter-community edges, in which *d* is the average degree of nodes. In summary, in each iteration, the algorithm needs O((*nK + m*)*K*) floating-point operations to conduct NMF and O(*n + pm + pd*) floating-point operations to modify the network topology. Thus, the computational complexity of our framework is near linear with the size of the network (*n* or *m*).

Besides, the three components in our framework, i.e., NMF, Connection Strategy and Disconnection Strategy, are all easily to be parallelized and distributed. To be specific, there are many parallel algorithms on NMF proposed recently^25^,^26^. The computation of the nodes' and edges' entropy in the Connection Strategy can be parallel implemented on multiple machines. And also, the operations in the Disconnection Strategy do not require complex calculations, and hence can be done quickly by only one machine.

To sum up, based on the above discussions our new semi-supervised framework may have the potential to be applied to real large-scale problems.

## Figures and Tables

**Table 1:** Real-world networks used here

Datasets	*n*	*m*	*K*	Descriptions
Karate	34	78	2	Zachary's karate club^20^
Dolphins	62	159	2	Dolphin social network^16^
Friendship6	68	220	6	High school friendship network^17^
Friendship7	68	220	7	High school friendship network^17^
Polbooks	105	441	3	Books about US politics^21^
Football	115	613	12	American College football^4^
Polblogs	1,490	16,718	2	Blogs about politics^23^
Baylor	12,803	679,817	7	Facebook networks at Baylor University^24^
USF	13,377	321,214	7	Facebook networks at University of San Francisco^24^
Syracuse	13,653	543,982	7	Facebook networks at Syracuse University^24^
Temple	13,686	360,795	7	Facebook networks at Temple University^24^
Northeastern	13,882	381,935	7	Facebook networks at Northeastern University^24^
UCSD	14,948	443,221	7	Facebook networks at University of California, San Diego^24^
UC	16,808	522,147	7	Facebook networks at University of California^24^
UVA	17,196	789,321	7	Facebook networks at University of Virginia^24^
USC	17,444	801,853	7	Facebook networks at University of Southern California^24^
NYU	21,679	715,715	7	Facebook networks at New York University^24^
UCLA	20,467	747,613	7	Facebook networks at University of California, Los Angeles^24^
Maryland	20,871	744,862	7	Facebook networks at University of Maryland^24^

## References

[b1] Malliaros F. D. & Vazirgiannis M. Clustering and community detection in directed networks: A survey. Phys. Rep. 533, 95–142; 10.1016/j.physrep.2013.08.002 (2013).

[b2] Fortunato S. Community detection in graphs. Phys. Rep. 486, 75–174; 10.1016/j.physrep.2009.11.002 (2010).

[b3] Palla G., Derényi I., Farkas I. & Vicsek T. Uncovering the overlapping community structure of complex networks in nature and society. Nature 435, 814–818; 1038/nature03607 (2005). 15944704 10.1038/nature03607

[b4] Girvan M. & Newman M. E. J. Community structure in social and biological networks. Proc. Natl. Acad. Sci. 99, 7821–7826; 10.1073/pnas.122653799 (2002). 12060727 PMC122977

[b5] Newman M. E. J. Detecting community structure in networks. Eur. Phys. J. B-Condensed Matter Complex Syst. 38, 321–330; 10.1140/epjb/e2004-00124-y (2004).

[b6] Nadakuditi R. R. & Newman M. E. J. Graph Spectra and the Detectability of Community Structure in Networks. Phys. Rev. Lett. 108, 188701; 10.1103/PhysRevLett.108.188701 (2012). 22681123

[b7] Decelle A., Krzakala F., Moore C. & Zdeborová L. Inference and Phase Transitions in the Detection of Modules in Sparse Networks. Phys. Rev. Lett. 107, 65701; 10.1103/PhysRevLett.107.065701 (2011). 21902340

[b8] Zhang Z.-Y., Sun K.-D. & Wang S.-Q. Enhanced Community Structure Detection in Complex Networks with Partial Background Information. Sci. Rep. 3; 10.1038/srep03241 (2013). PMC4894381 24247657

[b9] Eric E. & Rachael M. A spin-glass model for semi-supervised community detection. Paper presented at the 26th AAAI Conference on Artificial Intelligence, TorontoOntarioCanada. AtlantaGeorgiaUSA: AAAI Press. (2012, July 22–26).

[b10] Allahverdyan A. E., Ver Steeg G. & Galstyan A. Community detection with and without prior information. EPL (Europhysics Lett.) 90, 18002; 10.1209/0295-5075/90/18002 (2010).

[b11] Ma X., Gao L., Yong X. & Fu L. Semi-supervised clustering algorithm for community structure detection in complex networks. Phys. A Stat. Mech. its Appl. 389, 187–197; 10.1016/j.physa.2009.09.018 (2010).

[b12] Moore C., Yan X., Zhu Y., Rouquier J.-B. & Lane T. Active Learning for Node Classification in Assortative and Disassortative Networks. Paper presented at the 17th ACM SIGKDD international conference on Knowledge discovery and data mining, pages 841–849. New YorkNYUSA: ACM Press. (10.1145/2020408.2020552) (2011).

[b13] Leng M., Yao Y., Cheng J., Lv W. & Chen X. Active Semi-supervised Community Detection Algorithm with Label Propagation. Paper presented at the 18th International Conference for Database Systems for Advanced Applications, WuhanChina. BerlinGermany:Springer Press. (10.1007/978-3-642-37450-0_25) (2013, April 22–25).

[b14] Strehl A. & Ghosh J. Cluster ensembles---a knowledge reuse framework for combining multiple partitions. J. Mach. Learn. Res. 3, 583–617; 10.1162/153244303321897735 (2003).

[b15] Lancichinetti A., Fortunato S. & Radicchi F. Benchmark graphs for testing community detection algorithms. Phys. Rev. E 78, 46110; 10.1103/PhysRevE.78.046110 (2008). 18999496

[b16] Lusseau D. & Newman M. E. J. Identifying the role that animals play in their social networks. Proc. R. Soc. London. Ser. B Biol. Sci. 271, S477–S481; 10.1098/rsbl.2004.0225 (2004). PMC1810112 15801609

[b17] Xie J., Kelley S. & Szymanski B. K. Overlapping community detection in networks: The state-of-the-art and comparative study. ACM Comput. Surv. 45, 43; 10.1145/2501654.2501657 (2013).

[b18] Psorakis I., Roberts S., Ebden M. & Sheldon B. Overlapping community detection using bayesian non-negative matrix factorization. Phys. Rev. E 83, 66114; 10.1103/PhysRevE.83.066114 (2011). 21797448

[b19] Wang D., Li T., Zhu S. & Ding C. Multi-document summarization via sentence-level semantic analysis and symmetric matrix factorization. Paper presented at the 31st annual international ACM SIGIR conference on Research and development in information retrieval, pages 307–314. New YorkNYUSA: ACM Press. ((10.1145/1390334.1390387) (2008).

[b20] Zachary W. An Information Flow Modelfor Conflict and Fission in Small Groups. J. Anthropol. Res. 33, 452–473 (1977).

[b21] Newman M. E. J. Modularity and community structure in networks. Proc. Natl. Acad. Sci. 103, 8577–8582; 10.1073/pnas.0601602103 (2006). 16723398 PMC1482622

[b22] Zhang Z.-Y. Community structure detection in complex networks with partial background information. EPL (Europhysics Lett.) 101, 48005; 10.1209/0295-5075/101/48005 (2012). PMC4894381 24247657

[b23] Adamic L. A. & Glance N. The political blogosphere and the 2004 US election: divided they blog. Paper presented at the 3rd international workshop on Link discovery, pages 36–43. New YorkNYUSA: ACM Press. ((10.1145/1134271.1134277) (2005).

[b24] Traud A. L., Mucha P. J. & Porter M. A. Social structure of Facebook networks. Phys. A Stat. Mech. its Appl. 391, 4165–4180; 10.1016/j.physa.2011.12.021 (2012).

[b25] Liu C., Yang H., Fan J., He L.-W. & Wang Y.-M. Distributed Nonnegative Matrix Factorization for Web-scale Dyadic Data Analysis on Mapreduce. Paper presented a the 19th Int. Conf. World Wide Web, pages 681–690. New YorkNYUSA: ACM Press. (10.1145/1772690.1772760) (2010).

[b26] Demmel J. W., Heath M. T. & Van Der Vorst H. A. Parallel numerical linear algebra. Acta Numer. 2, 111–197 (1993).

